# Preparation of organotypic brain slice cultures for the study of Alzheimer’s disease

**DOI:** 10.12688/f1000research.14500.2

**Published:** 2018-06-27

**Authors:** Cara L. Croft, Wendy Noble

**Affiliations:** 1 Center for Translational Research in Neurodegenerative Disease, Department of Neuroscience, University of Florida, Gainesville, FL, 32610, USA; 2 Department of Basic and Clinical Neuroscience, Maurice Wohl Clinical Neuroscience Institute, Institute of Psychiatry, Psychology & Neuroscience, King's College London, London, SE5 9RX, UK

**Keywords:** Organotypic brain slice culture, neurodegeneration, amyloid-β, tau, Alzheimer’s disease, transgenic mice, reduction

## Abstract

Alzheimer's disease, the most common cause of dementia, is a progressive neurodegenerative disorder characterised by amyloid-beta deposits in extracellular plaques, intracellular neurofibrillary tangles of aggregated tau, synaptic dysfunction and neuronal death.

Transgenic rodent models to study Alzheimer’s mimic features of human disease such as age-dependent accumulation of abnormal beta-amyloid and tau, synaptic dysfunction, cognitive deficits and neurodegeneration. These models have proven vital for improving our understanding of the molecular mechanisms underlying AD and for identifying promising therapeutic approaches. However, modelling neurodegenerative disease in animals commonly involves aging animals until they develop harmful phenotypes, often coupled with invasive procedures.

We have developed a novel organotypic brain slice culture model to study Alzheimer’s disease using 3xTg-AD mice which brings the potential of substantially reducing the number of rodents used in dementia research from an estimated 20,000 per year. Using a McIllwain tissue chopper, we obtain 36 x 350 micron slices from each P8-P9 mouse pup for culture between 2 weeks and 6 months on semi-permeable 0.4 micron pore membranes, considerably reducing the numbers of animals required to investigate multiple stages of disease. This tractable model also allows the opportunity to modulate multiple pathways in tissues from a single animal. We believe that this model will most benefit dementia researchers in the academic and drug discovery sectors.

We validated the slice culture model against aged mice, showing that the molecular phenotype closely mimics that displayed *in vivo*, albeit in an accelerated timescale. We showed beneficial outcomes following treatment of slices with agents previously shown to have therapeutic effects *in vivo, *and we also identified new mechanisms of action of other compounds. Thus, organotypic brain slice cultures from transgenic mouse models expressing Alzheimer’s disease-related genes may provide a valid and sensitive replacement for *in vivo* studies that do not involve behavioural analysis.

Research highlights ***Scientific benefits:***

- Neurodegeneration in AD is progressive and age-dependent and therefore many experiments rely upon animals developing a moderate to severe phenotype prior to assessment of disease parameters
- Aging slices in a culture dish obviates this requirement, as well as providing an *in vitro* system in which all neural cell types are present, and functional and anatomical connectivity is retained

***3Rs benefits:***

- Here we describe methods of culturing pups from 3xTg-AD mice. A McIllwain tissue chopper is used to obtain 350 µm slices from postnatal day 8-9 mice that are cultured for 2-weeks to 6 months on 0.4 µm pore semipermeable membranes. Brain slice cultures can be used as an alternative to some *in vivo* experiments thereby reducing the numbers of animals required for such studies
- Disease phenotypes are accelerated in brain slice culture models so equivalent data can be obtained from neonatal mice in 1 month of *in vitro* experiments rather than 12 months of aging *in vivo* from fewer total mice
- 36 brain slices can be cultured from a single mouse brain, so studies can be refined as it is possible to assess several parameters or disease-modifying agents in a single brain thereby minimising biological variation

***Practical benefits:***

- This model is considerably more time and cost effective when compared to *in vivo* studies

***Current applications:***

- The 3xTg-AD slice culture model is suitable for biochemical and immunohistochemical research into Alzheimer’s disease and related tauopathies

***Potential applications:***

- Long-term organotypic brain slice cultures have the potential for use in all aspects of neuroscience
- Cultures from other tissues will have utility for many other fields of biological and biomedical research

## Introduction

Alzheimer’s disease (AD), the most common cause of dementia, currently affects around 35 million people worldwide and carries huge societal and economical costs. AD is a multi-factorial disease with two major pathological hallmarks; extracellular plaques composed of β-amyloid (Aβ) and intracellular neurofibrillary tangles containing aggregated post-translationally modified tau. The only available treatments for AD target the symptoms of disease, but not disease course. Intensive research efforts are ongoing to better understand the biological causes of disease so that effective disease-modifying therapies can be developed.

Perhaps the most accepted models for AD research are transgenic rodents that express wild-type or mutant human AD-associated genes and recapitulate key molecular phenotypes of AD. Mice are generally one of the best accepted animal models in neuroscience research since there is significant homology between the human and mouse genome, mice have a relatively short life span, well-defined genetic backgrounds, are amenable to further genetic manipulations, enable assessment of changes in behaviour, cognition, brain biochemistry and physiology during disease progression, and a battery of well-characterised tasks are available to study behaviour and cognition ^1^.

Our estimates, based on a PubMed search using the terms “Alzheimer’s + transgenic + mouse” and our evaluation that an average of 30 mice for each of the 700 papers published, suggest that over 20,000 wild-type and transgenic mice were used in AD research in 2017. Due to the age-related neurodegenerative nature of the disease, this research often involves aging several cohorts of mice to observe disease progression. Allowing mice expressing AD-related genes to reach the terminal stages can result in severe phenotypes, and some studies are coupled with invasive procedures such as advanced live imaging or collection of interstitial or cerebrospinal fluids.

Alternatives to *in vivo* AD research in mammalian systems include rodent and human cell lines manipulated to express genes of interest, however these can be criticised for lacking key features of differentiated post-mitotic neurons and can be prone to artefacts resulting from protein over-expression. Dissociated neural cell cultures are commonly used as a readily tractable model in which pathways of interest can be manipulated, however even co-culture systems do not completely replicate the complex connections between different neural cell types and the brain vasculature, and they cannot model the synaptic and anatomical connectivity of mammalian brain. The latter is also true for neural cells derived from human induced pluripotent stem cells (iPSCs). Recent reports using iPSC-derived neurons also highlights the extensive time in culture required before even subtle disease relevant changes are observed in these human neural cells ^2^.

Organotypic brain slice cultures are a well-established technique. Slice cultures maintain a three-dimensional organisation with the preservation of cytoarchitecture and cell populations, and are an accessible system lending their application to electrophysiology, morphology and biochemical analyses ^3– 5^. The interface-slice culture method established by Stoppini and colleagues in 1991 ^6^ is the most common method to culture brain sections *ex vivo*. This relatively simple method cultures brain tissue explants from neonatal mice/rats on a porous membrane insert that acts as an interface between the humidified incubator atmosphere and the culture medium that provides nutrition ^6^. Cultures can be maintained for several weeks in culture after explant and continue to develop and mature once plated ^7, 8^. In addition, 36 slices containing the cortex and hippocampus can be prepared from one postnatal mouse brain allowing multiple variables to be tested or manipulated in tissue from the same mice, thereby considerably reducing the number of mice required as well as minimising experimental variation. Importantly, slice cultures are prepared from neonatal (P8-9) mice which removes the need to age mice until stages where they develop adverse phenotypes and avoids aging several groups of mice to different disease stages for these studies. This method requires only the maintenance of small breeding colonies which also minimises the inherent costs.

AD researchers are beginning to embrace organotypic brain slice culture models, with recent papers describing AD-relevant changes in slice cultures prepared from mice that overexpress amyloid precursor protein or are seeded with Aβ, which show some accumulation of Aβ and synaptic alterations ^9– 11^. Others have shown that slice cultures prepared from mice that overexpress human tau can accumulate phosphorylated and some sarkosyl-insoluble tau ^11, 12^. We recently demonstrated that slice cultures prepared from 3xTg-AD mice ^13^ overproduce Aβ, accumulate somatodendritic and synaptic phosphorylated tau at an accelerated rate compared to 3xTg-AD mice ^14^, allowing study of Aβ-tau interactions and AD disease pathways *ex vivo*.

The utility of slice cultures for drug discovery efforts has previously been reviewed ^15^, and we have validated 3xTg-AD slice cultures for this purpose by showing that the effects of disease-modifying compounds observed *in vivo* can be recapitulated in slice culture ^16^. We also identified novel targets for compounds, further demonstrating the usefulness of slice cultures for therapeutic development. In this paper, we provide detailed methods for the preparation of organotypic brain slice cultures for the study of AD and we discuss the advantages of this model system in terms of the 3Rs in AD research, most specifically in reducing mouse numbers. We believe that this model system will be of most benefit to researchers in the neurodegeneration field, who are either focussed on understanding the biological mechanisms underpinning disease or who aim to screen and test the efficacy of novel disease-modifying therapeutics.

## Methods overview

### Animals

3xTg-AD mice were obtained under a material transfer agreement from Professor Frank LaFerla (University of California Irvine, USA) and maintained as a breeding colony at King’s College London. 3xTg-AD mice express mutant human PS1 (M146V), APP (Swe, K670N, M671L) and tau (P301L) transgenes ^13^. Wild-type (WT) mice of an identical background strain (F2 hybrid: C57BL/6J and 129S1/SvImJ) were maintained as background controls. All housing and experimental procedures were carried out in compliance with the local ethical review panel of King’s College London under a UK Home Office project licence held in accordance with the Animals (Scientific Procedures) Act 1986 and the European Directive 2010/63/EU. Male and female mice were used in this study. After weaning, mice were housed in single sex groups in standard 40 x 25 x × 12 cm plastic cages. Bedding consisted of kiln dried aspen shavings and paper sizzle nest material (Datesand Ltd, Manchester, UK). Water and food were available (Picolab rodent diet 20; #5053; Lab Diet, St Louis, MO) *ad libitum*. Animals were housed at 19–22°C, humidity 55%, 12 h:12 h light: dark cycle with lights on at 07:30. Cages were cleaned once every two weeks, with mice handled by the tail by experienced animal care staff to transfer them between cages.

### Slice culture preparation

Organotypic brain slice cultures were prepared from postnatal day (P) 8-9 3xTg-AD and background control wild-type mice as previously described ^11, 14^. Briefly, pups were culled by decapitation in accordance with the UK Animals in Scientific Procedures Act (1986). Brains from pups were bisected into hemi-brains by a single cut along the midline. The cerebellum, thalamus and brainstem were removed and discarded to leave the cortex, hippocampus and connecting areas. These were kept in ice-cold dissection buffer (1.25 mM KH _2_PO _4_ pH 7.4, 124 mM NaCl, 3 mM KCl, 8.19 mM MgSO _4_, 2.65 mM CaCl _2_, 3.5 mM NaHCO _3_, 10 mM glucose, 2 mM ascorbic acid, 39.4 µM ATP in ultrapure H2O, sterile filtered (0.2 µm)) with constant oxygenation throughout the preparation procedure. 350 µm coronal slices were cut using a McIlwain Tissue Chopper (Stoelting Europe, Ireland). Eighteen slices from each hemi-brain were collected and 3 consecutive slices per well were positioned on interface-style Millicell culture inserts (Millipore (UK) Ltd.) in 6 well culture plates (ThermoFisher Scientific, UK) containing 1 mL of sterile slice culture medium (Basal medium eagle (BME), 26.6 mM HEPES, pH 7.1, 19.3 mM NaCl, 5 mM NaHCO _3_, 511 µM ascorbic acid, 40 mM glucose, 2.7 mM CaCl _2_, 2.5 mM MgSO _4_, 1% (v/v) GlutaMAX (Life Technologies, Paisley, UK), 0.033% (v/v) insulin, 0.5% (v/v) penicillin/streptomycin (Life Technologies), in ultrapure H _2_O, sterile filtered (0.2 µm), plus 25% (v/v) heat inactivated horse serum (ThermoFisher, UK). Three hours after plating, the culture medium was removed by aspiration and replaced with 1 mL of pre-warmed fresh sterile culture medium. Brain slices were incubated at 37°C and the culture medium was changed from the bottom of each well every 2 to 3 days. Slices are maintained for a minimum of 14 days *in vitro* prior to treatment/harvesting.

### Sample preparation and analysis

Slice cultures can be pharmacologically or genetically modified using a number of methodologies. These methods are out of the scope of this publication but have previously been published by ourselves and others (for example, 9– 11, 14, 16, 17).

Organotypic brain slice cultures can be fixed on their membrane inserts in 4% PFA for 4 h and stained according to Croft *et al*. ^14, 18^. In brief, slice cultures are cut whilst still on their membranes and then treated as free-floating sections. Slice cultures are permeabilised for 18h in 0.5% Triton X-100 at 4°C and then blocked in 20% bovine serum albumin (BSA) for 4h at RT. Slice cultures are incubated with primary antibodies overnight at 4°C in 5% BSA, washed and then incubated with fluorophore-coupled secondary antibodies for 4h at ambient temperature. Slice cultures are washed a final time before mounting on slides with fluorescent mounting medium (Dako Ltd., Ely, UK) prior to imaging. 

Alternatively, tissue can be lysed for subcellular fractionation and/or biochemical analysis as described by us and others ^10, 11, 14, 16, 17^. To prepare lysates for immunoblotting, slice culture medium is aspirated and slices washed once with ice-cold PBS. Slices are collected via scraping into ice-cold PBS. Cellular matter is pelleted by centrifugation at 7,000 g (av) for 30 seconds at ambient temperature. The supernatant is discarded and tissue pellets lysed in 100 μL ice-cold extra strong lysis buffer (10 mM Tris-HCl (pH 7.5), 0.5% (w/v) sodium dodecyl sulphate (SDS), 20 mM sodium deoxycholate, 1% (v/v) Triton-X-100, 75 mM sodium chloride, 10 mM ethylenediaminetetraacetic acid (EDTA), 2 mM sodium orthovanadate, 1.25 mM sodium fluoride) and protease inhibitor cocktail for mammalian tissues (Roche Diagnostics, UK). The suspension is then sonicated briefly (10 seconds) using a Vibra-Cell™ probe sonicator to improve sample handling. Slice lysates are centrifuged at 23,000 g(av) for 20 minutes at 4°C and the supernatant collected. The protein content of the slice lysates can be determined using a BCA protein assay (Pierce Endogen, Rockford, USA) and protein concentration normalised prior to immunoblotting or ELISA. Slice lysates are mixed with an equal volume of 2x sample buffer before immunoblotting.

Culture medium can also be collected for analysis of its components, as we recently described for tau and Aβ ^14^. Slice culture medium is replaced with Hank’s Balanced Salt Solution (HBSS; Life Technologies Ltd). HBSS is collected from the slice cultures and centrifuged at 12,000g for 10 min at 4°C to remove cell debris. Protein content in HBSS can be determined by ELISA by standard or sandwich ELISA.

## Full protocol for the model development

### Equipment required

McIllwain Tissue Chopper (RRID:SCR_015798; Mickle Laboratory Engineering Co. Ltd., Surrey, UK)

Stereomicroscope for tissue dissection

Chopping discs (Product code: 752TC-CT; Campden Instruments Ltd., Loughborough, UK)

Blades (Product code: TC752-1; Campden Instruments Ltd., Loughborough, UK)

55mm diameter ashless filter paper (Product code: WHA1442055; Merck, UK)

Flat (cover glass) forceps (Product code: 11074-02; Fine Science Tools, Heidelberg, Germany)

Cohan-Vannas Spring Scissors (Product code: 15000-02; Fine Science Tools, Heidelberg, Germany)

Mayo scissors (Product code: 14010-17; Fine Science Tools, Heidelberg, Germany)

Fine scissors, sharp (Product code: 14060-11; Fine Science Tools, Heidelberg, Germany)

Moria MC17C perforated spoon-mini (Product code: 10370-19; Fine Science Tools, Heidelberg, Germany)

Mini hippocampal dissection tool (Product code: 10099-12; Fine Science Tools, Heidelberg, Germany)

Cell culture treated 6-well plates (Product code: 140675; ThermoFisher Scientific, UK)

Cell culture treated 10cm dish (Product code: 172931; ThermoFisher Scientific, UK)

PTFE 30mm tissue culture inserts 0.4μm (Product code: PICM03050; Millipore, Fisher Scientific, UK)

Pasteur pipettes – sterile – individually wrapped (Product code: Z350621-400EA; Merck, UK)

30ml Pyrex beaker (Produce code: CLS100030; Merck, UK)

Fine paintbrush

Carbogen (95% Oxygen / 5% Carbon Dioxide)

Standard tissue culture incubator (37°C / 5% Carbon Dioxide)

### Buffers and culture medium

Slice Culture Dissection buffer: 1.25 mM KH _2_PO _4_ pH 7.4, 124 mM NaCl, 3 mM KCl, 8.19 mM MgSO _4_, 2.65 mM CaCl _2_, 3.5 mM NaHCO _3_, 10 mM glucose, 2 mM ascorbic acid, 39.4 µM ATP in ultrapure H _2_O, sterile filtered (0.2 µm).

Slice culture medium: Basal medium eagle (BME), 26.6 mM HEPES, pH 7.1, 19.3 mM NaCl, 5 mM NaHCO _3_, 511 µM ascorbic acid, 40 mM glucose, 2.7 mM CaCl _2_, 2.5 mM MgSO _4_, 1% (v/v) GlutaMAX (Life Technologies, Paisley, UK), 0.033% (v/v) insulin, 0.5% (v/v) penicillin/streptomycin (Life Technologies), in ultrapure H _2_O, sterile filtered (0.2 µm), plus 25% (v/v) heat inactivated horse serum (ThermoFisher, UK).

### Methods

*Brain extraction:*

**Figure 1.**
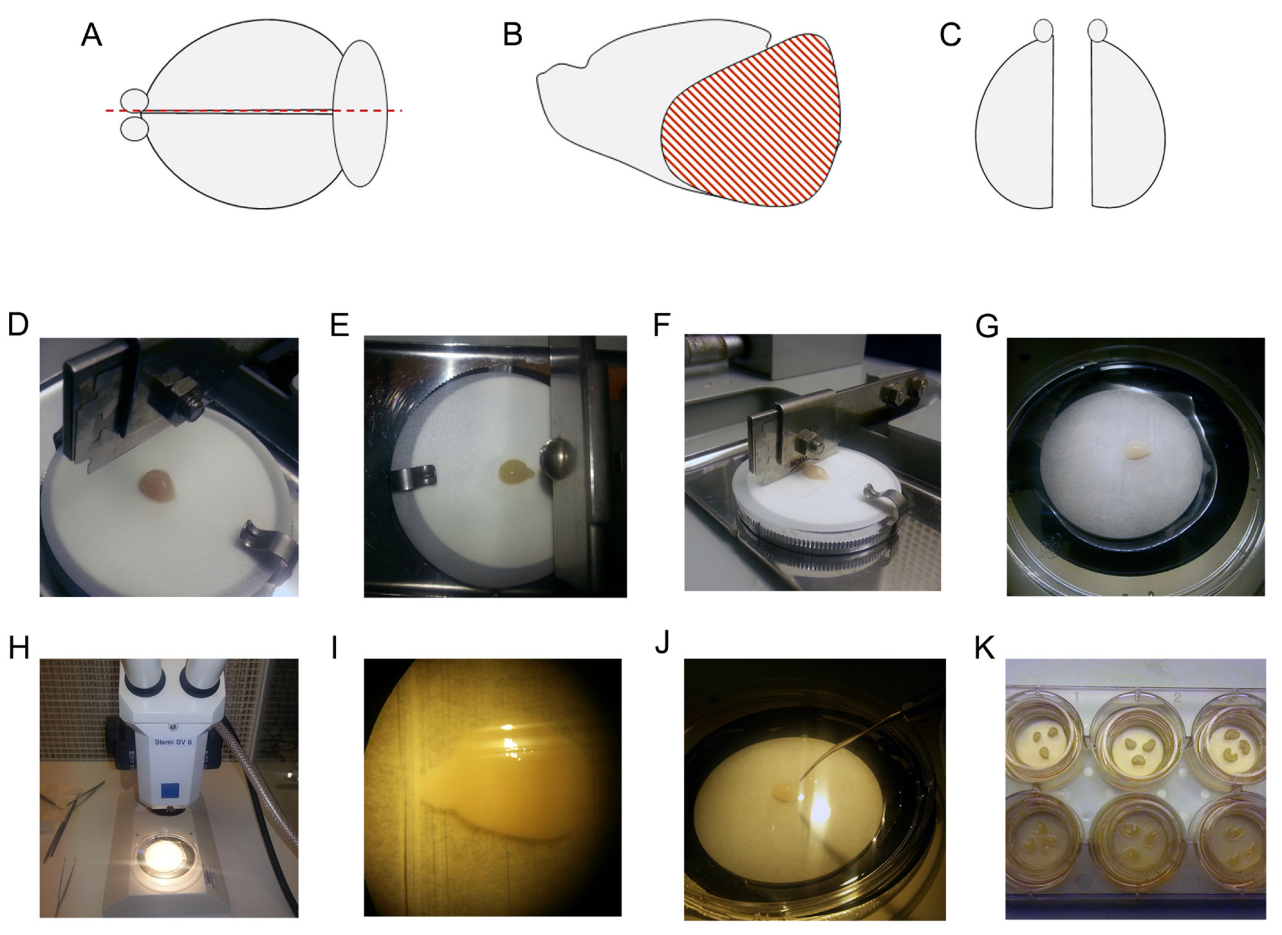
Preparation of organotypic brain slice cultures. ( **A**) After removal from the skull, brains are bisected along the midline using a razor blade. ( **B**) The thalamus, cerebellum and brain stem are removed using the hippocampal dissection tool leaving the cortex, hippocampus and connected brain regions. ( **C**) Two hemi-brains are kept in oxygenated dissection buffer throughout the procedure; one hemi-brain is stored whilst the other is processed. ( **D**) A hemi-brain is placed on dampened filter paper on the cutting surface of a McIlwain tissue chopper. ( **E**– **F**) 350 µm coronal slices are cut. ( **G**– **J**) Slice cultures are sequentially separated under a dissection microscope using a hippocampal dissection tool. ( **K**) Three slices are plated per well on Millipore membrane inserts in 6 well plates. Three consecutive slices can be placed in each well or slices plated randomly depending on experimental needs. Cultures are maintained by replacing the culture medium every 2–3 days.

Experiments are performed under sterile conditions with tools sterilised by autoclaving prior to use. 70% EtOH is used to sterilize equipment and surfaces throughout the experiment. Postnatal day 8 or 9 WT or 3xTg-AD mice are used ( Figure 1A–C).

1. Pups are decapitated using Mayo scissors and death confirmed.
2. Heads are transferred to a 10cm tissue culture dish containing oxygenated ice-cold dissection buffer.
3. Fine scissors are used to remove hair and skin, cutting anteriorly from the base of the skull along the midline, revealing the brain and skull.
4. Small spring scissors are used to carefully cut through the midline of the skull.
5. The brain is bisected through the midline while remaining in the skull using a razor blade.
6. The hippocampal dissection tool is used to cleanly remove the remove brainstem, cerebellum and thalamus which are discarded. The cortex, hippocampus and associated regions remain intact.
7. The remaining tissue is removed from the skull and transferred to a glass beaker containing oxygenated dissection buffer.
8. Repeat for the other hemi-brain. One hemi-brain will remain in dissection buffer, regularly re-oxygenated, while the other is processed.

*Slice culture preparation ( Figure 1D–K):*

9. 1 mL of slice culture medium is added to each well of a 6-well culture plate. Flat cover glass forceps are used to add a Millicell culture insert into each well. The plate is returned to a 37°C incubator to ensure that culture medium is pre-warmed before slices are plated.
10. A plastic chopping disc and fresh filter paper is placed on the cutting stage of a McIlwain tissue chopper. Three to four drops of dissection solution are used to dampen the filter paper and allow the hemi-brain to remain in place.
11. A hemi brain is placed onto the filter paper and oriented for coronal sectioning (the front of the brain on the right-hand side).
12. A drop of dissection solution is added to the brain to prevent the cutting blade from sticking.
13. The section size on the McIllwain tissue chopper is adjusted to 350 µm. The blade should be manually positioned adjacent to the frontal region of the brain.
14. The tissue chopper is started - the automated razor blade will cut 350 µm sections until manually switched off. The speed of cutting can be adjusted if necessary.
15. The hemi-brain, remaining on the filter paper, is transferred to a fresh 10cm dish containing oxygenated dissection solution, and the dish is placed under a dissection microscope.
16. Slices are manually separated using the hippocampal dissection tool, taking care to avoid ripping tissue. Using a plastic Pasteur pipette, individual slices are transferred to culture inserts in 6-well plate.
17. From the leading edge (frontal cortex), three consecutive slices are positioned in each culture insert. Each hemi-brain is sectioned into 18 slices, equivalent to one 6-well plate. Alternatively, slices can be plated randomly and distributed throughout wells allowing the study of frontal, middle and rostral sections within each well.
18. Care should be taken to ensure that slices do not overlap or make contact with the sides of the insert. A fine paintbrush can be used to move the slices and to ensure that no areas of the slices are folded or wrinkled.
19. Excess dissection solution is removed from the slice culture inserts, and the 6-well plate is returned to an incubator and maintained in humid conditions at 37°C with 5% CO _2_.

*Slice culture maintenance:*

20. In sterile conditions, approximately three hours after plating a glass Pasteur pipette is used to aspirate culture medium. 1 mL pre-warmed fresh sterile culture medium is then added.
21. Brain slices are incubated in humid conditions at 37°C with 5% CO _2_. Culture medium is replaced every 2 to 3 days, taking care not to move the inserts within each well. Any excess medium which collects in the insert is also removed during media changes, taking care not to disturb the slices.
22. Slice cultures can be analysed from 14 days after plating, at which time lactate dehydrogenase release should have returned to basal levels ^11^.

### NOTES: Practical considerations and tips

• P8-P9 pups are used in this method, however other ages of pups are described in publications from other groups. It is likely that some optimisation may be required depending on the strain or transgenic line of mice being used.
• Slice cultures are initially white in colour, but become translucent after 7 to 10 days in culture. White tissue remaining at this point is likely to signify unhealthy or dead tissue.
• It is important to cleanly remove all of the thalamus, cerebellum and brainstem during dissection since these are detrimental to slice survival under these conditions. These tissues can be cultured but using alternative protocols.
• It is important that excess dissection buffer is removed from the slice cultures once they have been plated since prolonged exposure to this buffer in culture can affect slice health.

## Results

### Characterisation of the model: Organotypic brain slice cultures from 3xTg-AD mice recapitulate molecular features of AD and show an accelerated disease phenotype compared to *in vivo*

We have previously characterised organotypic brain slice cultures prepared from 3xTg-AD mice in comparison to brain from aged *in vivo* 3xTg-AD mice ^14^. We examined abnormalities in β-amyloid and tau that accumulate in AD brain. We found that 3xTg-AD slice cultures show an accelerated development of highly phosphorylated and oligomeric/64kDa tau species, some of which redistributed to synaptic compartments by 28 days *in vitro (DIV)*. Similar changes *in vivo* are typically observed from 12 months of age. An accelerated accumulation of potentially pathogenic Aβ species were also observed in brain slice cultures from 3xTg-AD mice, with significantly increased Aβ1-42 levels detected at 28 *DIV* in slices. In comparison, we could only detect significant changes in Aβ1-42 amounts in 3xTg-AD brain in 12-month old mice. Thus, disease-associated protein species show an accelerated accumulation in long-term brain slice cultures in comparison to *in vivo*. Using differential centrifugation approaches we were also able to show the differential accumulation of phosphorylated and dephosphorylated tau species in synaptic compartments and at membranes, in agreement with previous reports using human tissue and primary cell cultures ^19, 20^. Table 1 provides a summary of molecular changes in the slice culture model in comparison to findings made using tissue from aged 3xTg-AD mice. Primary data is available here ( Dataset 1).

**Table 1.** Biochemical and pathological features of 3xTg-AD organotypic brain slices in comparison to *in vivo* 3xTg-AD brain and AD brain. Primary references are shown. AD: Alzheimer’s disease; DIV: days *in vitro*.

Feature	AD brain	3xTg-AD mice *in vivo*	3xTg-AD *ex vivo* brain slice
**Tau phosphorylation**	Increased at many sites ^21^	Increased in 12–15-month old mice ^13^ in hippocampus but not cortex ^14^	Increased at Ser202 and Ser396/404 by 28 DIV ^14, 16^
**High molecular weight** **(HMW) tau/tau aggregation**	Tau aggregates in characteristic neurofibrillary pathology ^22^	HMW and sarkosyl-insoluble tau aggregates by 12 months of age ^14^, tau aggregates and NFTs at 18 months of age ^13^.	64kDa tau at 21 and 28 DIV ^14^
**Aβ**	Increased Aβ deposition in hallmark plaques ^23, 24^	Increased Aβ-42 at 6–12 months of age ^13, 14, 25^, plaques from 6 months of age ^13^	Increased Aβ-42 by 14 DIV ^14^
**Synaptic protein loss**	Loss of PSD-95 and synaptophysin ^26, 27^	Loss of PSD-95 and synaptophysin at 13 months of age ^28^, but not 12 months of age ^14^	No loss of PSD-95 or synaptophysin ^14^
**Synaptic tau**	Tau in AD and control synapses, but phosphorylated tau species only in AD synapses ^29^	Tau at synapses transiently increased at 1–2 month of age, then returning to control levels until 12 months of age ^14^	Tau at synapses transiently increased at 14 DIV, then returning to normal levels until 28 DIV ^14^
**Synaptic APP**	APP not increased in AD synapses ^30^	Increased APP in synapses at 1–2 months of age, but not at later ages up to 12 months ^14^	No change in synaptic APP ^14^

There is also a great deal of versatility in the methods that can be used to assess disease changes in this model. We have confirmed that methods including, but not limited to the following, can be used with slice cultures; biochemical changes can be assessed by ELISA or immunoblotting, slice cultures can be examined by immunohistochemistry and confocal microscopy, sufficient material is present to allow differential centrifugation to enrich cell compartments such as synaptosomes, membrane and cytosol. Additionally, cell death can be measured using lactate dehydrogenase assays and the release of disease-associated proteins into culture medium can be quantified ^14, 31^. Others have used imaging methods to describe the anatomy of slice cultures ^6, 7, 32^ and have also shown that brain slice cultures are amenable to ultrastructural analysis ^33^, live cell imaging ^34– 36^ and live calcium imaging ^12^.

### Validation of the model: Organotypic brain slice cultures can be used to assess acute pharmacological treatments and to determine drug targets

In addition to comparing molecular features of AD in slice cultures in comparison to *in vivo*, we also validated the use of slice cultures for studying the effects on tau phosphorylation of acute application of compounds in comparison to their reported effects in previously published *in vivo* studies.

Lithium chloride (LiCl) can inhibit activity of the prominent tau kinase, glycogen synthase kinase-3β (GSK-3) ^37^, which targets many of the tau residues known to be aberrantly phosphorylated in AD ^21^. Treatment of 12-month-old 3xTg-AD mice for 3 months with the GSK-3 inhibitor, LiCl, was shown to reduce phosphorylation of tau at Thr 181, Ser 202/Thr 205, Thr 231, and Thr 212/Ser 214, but not Ser 396/404 ^38^. We found that application of LiCl for 4 h to 3xTg-AD brain slice cultures at 28 days days *in vitro* (DIV) resulted in significantly reduced tau phosphorylation at the Ser396/404 and Ser202/Thr205 epitopes, in addition to causing a subtle reduction in total tau amounts when compared to slices treated with control (NaCl). There was also a notable shift in the apparent molecular weight of tau in lysates from LiCl treated slice cultures, which is characteristic of reduced tau phosphorylation ^16^. Of interest, others have shown that okadaic acid induces hyperphosphorylation of tau in organotypic brain slice cultures prepared from TG APP_SweDI mice (SweDI; expressing APP harboring the Swedish K670N/M671L, Dutch E693Q, and Iowa D694N mutations; C57BL/6-Tg(Thy1-APPSwDutIowa)BWevn/Mmjax) ^17^. Thus, this platform is very amenable for the study of treatments that modulate tau phosphorylation.

Another potential therapeutic approach for AD is to use microtubule-stabilising agents to recover the loss of function, which occurs following the detachment of phosphorylated tau from the microtubule cytoskeleton ^37^. Neuroprotective effects of the peptide NAPVSIPQ have previously been reported in 12-month-old 3xTg-AD mice. Mice administered NAPVSIPQ for 3 months showed reduced phosphorylation of tau at Ser 202/Thr 205 and Thr 231, but not at Ser 202 alone. Treatment of 28 DIV 3xTg-AD slice cultures with 100 nM NAPVSIPQ for 24 h significantly reduced tau phosphorylation at the Thr231 epitope, but did not alter the total amount of tau when compared to control cultures ^16^, thus we find that equivalent treatment of 3xTg-AD organotypic brain slice cultures recapitulates previous *in vivo* findings conducted in 3xTg-AD mice ^38– 40^.

Primary data is available at https://www.ncbi.nlm.nih.gov/pmc/articles/PMC5547074/ ^16^.

We also used the slice culture model to identify novel tau-directed effects of BTA-EG _4_^16^, a compound that had previously shown Aβ-binding effects and synaptic protection ^41– 43^. A growing body of publications have further demonstrated the tractability of the slice culture system, including pharmacological manipulation of Aβ production ^10^ and tau aggregation ^12^, in addition to modulation of microglial composition to examine the phagocytic action of microglia on Aβ deposits ^33^.

These data suggest that potential therapeutic agents can be sensitively examined in organotypic brain slice culture models. Since a number of methods can be applied to study slice culture tissues, this system should be considered as a replacement for *in vivo* studies with molecular and cellular study parameters and when end-points do not include life-span or behavioural assessment. Certainly, in academic and industrial laboratories, slice cultures should provide an excellent system for medium throughput drug screening or range-finding studies.

Primary data for molecular changes in the slice culture model in comparison to findings made using tissue from aged 3xTg-AD mice Click here for additional data file. Copyright: © 2018 Croft CL and Noble W 2018 Data associated with the article are available under the terms of the Creative Commons Zero "No rights reserved" data waiver (CC0 1.0 Public domain dedication).

## Discussion

### Organotypic brain slice cultures can reduce the number of animals required for some *in vivo* studies

Depending on the nature of the experiment, one postnatal day 8 or 9 pup can provide an n=36 for immunohistochemical analysis, and a single well containing three slices can be combined to give n=12 for biochemical analysis or compound screening. For example, the preparation of slice cultures from only six postnatal pups would allow the opportunity to study 12 time-points in six different animals, a reduction in numbers of 91% in comparison to the 72 mice that would be required for an *in vivo* aging study. In addition, since multiple time points will be assessed in tissues from the same animals, experimental within-group variation is substantially reduced.

Take-up of this method within academic laboratories in the UK appears to be growing, however it is very difficult to accurately quantify the number of animals that have not been used as a result of researchers preparing slice cultures in preference to *in vivo* experimentation. Within our own laboratories, we estimate that our *in vivo* experimentation has reduced by approximately 20% as we train more researchers in the method of brain slice culture preparation. 

### An adaptable model for neurodegeneration research

While we have focussed on AD research in this article, organotypic brain slice cultures are equally suitable for research into a range of other neurodegenerative and neurological conditions, as well as for basic neuroscientific research (reviewed by 44). Slice cultures can also be prepared specifically from the hippocampus ^10^ or from other tissues such as spinal cord ^45^; the latter being used to investigate prion-like properties of mutant SOD1 proteins in amyotrophic lateral sclerosis. The technique is not limited to mice, rats are commonly used ^44^ and methods are emerging to allow long-term culture of human organotypic brain slice cultures ^46^. There are no major restrictions on uptake of this model since it requires only modest investment in terms of equipment provision. Training in tissue dissection and slicing may be beneficial, but the technique can readily be learned with practice.

### Limitations of this model

It is important to consider some of the limitations associated with organotypic slice cultures in general, and in the context of Alzheimer’s disease research. Firstly, we use a mouse line here that expresses genetic mutations in APP and PS1 that cause AD, alongside an FTD-causing tau mutation. It is unclear to what extent mice expressing FAD-causing genes recapitulate the more common sporadic forms. However, pathophysiological similarities between familial and sporadic AD suggests that familial models have, at least some, utility for investigating disease mechanisms. For example, recent work from the expansive DIAN-TU study highlights the similar progression and overlapping pathology between sporadic and familial AD, which further supports the use of familial models to understand sporadic AD ( https://www.alzforum.org/news/conference-coverage/dian-and-adni-data-say-familial-and-sporadic-ad-converge).

More generally, the limitations of slice cultures should be considered by users. These have been reviewed elsewhere ^44^, and will only be briefly mentioned here.

- Slice cultures do not have a functioning brain vasculature. In the context of neurodegeneration, this may mean that they are not the best system for studying changes in the neurovascular unit or those resulting from changes in cerebral blood flow. However, they likely still have advantages over dissociated co-culture primary cell culture for this purpose, and the lack of a blood-brain-barrier has advantages for drug screening.
- Slice cultures have been axotomised, and as a result show loss of target innervation. This may be somewhat ameliorated by the addition of nerve growth factors ^44^. We recommend a “resting period” of 2 weeks before analysing plated slices to allow many of the acute effects of axotomy to be resolved.
- Slice cultures develop, at least soon after plating, a layer of reactive astrocytes on their outer surface as a protective response to cutting ^34^. This may present difficulties for some analysis methods including imaging and patch clamping, although these can be overcome.
- Slice cultures become flattened over time (100–200µm), which is partially reduced by the addition of high concentrations of horse serum, but results in some disruption to their anatomical structure and electrophysiological properties ^44^.

## Conclusions

Here, we describe a detailed method for the preparation of long-term organotypic brain slice cultures from postnatal mice. We describe our work previously showing that slice cultures prepared from 3xTg-AD mice recapitulate important molecular and cellular features of *in vivo* disease development and the human disease phenotype. We also summarise the versatility of the model for drug discovery and the acute screening of compounds. Slice cultures show a significant acceleration in the timescale in which disease features develop, with relevant pathological changes observed at 28 days *in vitro* as opposed to 12 months *in vivo* in 3x-TgAD mice. We suggest that organotypic brain slice cultures can be used to replace several *in vivo* studies and that their widespread uptake could reduce the number of animals used in neurodegenerative disease research by 20–50%. This could be achieved if slice cultures were used in place of purely biochemical and immunohistological studies, and for experiments not reliant on behavioural outcomes.

## Data availability

The data referenced by this article are under copyright with the following copyright statement: Copyright: © 2018 Croft CL and Noble W

Data associated with the article are available under the terms of the Creative Commons Zero "No rights reserved" data waiver (CC0 1.0 Public domain dedication). 

Dataset 1: Primary data for molecular changes in the slice culture model in comparison to findings made using tissue from aged 3xTg-AD mice. DOI: 10.5256/f1000research.14500.d200832 ^20^

## Supplementary material

Supplementary File 1: ARRIVE checklist.
