# Supplementary material for: Preparation of organotypic brain slice cultures for the study of Alzheimer’s disease
Source: F1000Res. 2018 Jun 27;7:592. Originally published 2018 May 15. [Version 2] doi: 10.12688/f1000research.14500.2 (PMC5964634; doi:10.12688/f1000research.14500.2)
Supplement: Peer review history

# Peer review: Referee response for version 2

**DOI:** 10.5256/f1000research.16770.r35529

**Published:** 2018-06-28

**Authors:** Claire S. Durrant

**Affiliations:** 1 John van Geest Centre for Brain Repair, Department of Clinical Neurosciences, University of Cambridge, Cambridge, UK

The revised manuscript has clarified some important methodological points which will be of great use to future readers. I am happy to approve this version.

I have read this submission. I believe that I have an appropriate level of expertise to confirm that it is of an acceptable scientific standard.

# Peer review: Referee response for version 2

**DOI:** 10.5256/f1000research.16770.r35528

**Published:** 2018-06-27

**Authors:** Christian Humpel

**Affiliations:** 1 Laboratory of Psychiatry and Experimental Alzheimer’s Research, Medical University of Innsbruck, Innsbruck, Austria

I have read this submission. I believe that I have an appropriate level of expertise to confirm that it is of an acceptable scientific standard.

# Peer review: Referee response for version 1

**DOI:** 10.5256/f1000research.15785.r34402

**Published:** 2018-05-29

**Authors:** Susan C. Barnett

**Affiliations:** 1 Institute of Infection, Immunity and Inflammation, College of Medical, Veterinary and Life Sciences, University of Glasgow, Glasgow, UK

This is a well written clearly explained method for making slice cultures from 3xTg-AD mice for the study of Alzheimer’s disease. It is useful to have a step by step account with hints to describe this method as in general slice cultures can be difficult to reproduce and be consistent. There is a good description of how this method fulfils NC3Rs criteria.

 Minor points:

1. It would be very helpful to have a little more justification of why this is a novel method when many publications can be seen using slice cultures for Alzheimers research, even from transgenic mice.
2. It was a little bit confusing on the section stating: In brief, slice cultures are cut whilst still on their membranes and then treated as free-floating sections. Is this illustrated in Figure 1? If so could be made clearer?
3. Not convinced the n=12 from one pup statement is correct, as this would be replicates. N usually refers to different biological repeats.
4. It would be really useful to see example of the slices when validated, with examples of staining. Can microglia be seen and how good is the anatomy of the slices.
5. The LDH was not explained enough and perhaps the time course could have been shown.

**Are a suitable application and appropriate end-users identified?**

 Yes

**If applicable, is the statistical analysis and its interpretation appropriate?**

 Yes

**Are the 3Rs implications of the work described accurately?**

 Yes

I have read this submission. I believe that I have an appropriate level of expertise to confirm that it is of an acceptable scientific standard.

# Peer review: Referee response for version 1

**DOI:** 10.5256/f1000research.15785.r34019

**Published:** 2018-05-22

**Authors:** Christian Humpel

**Affiliations:** 1 Laboratory of Psychiatry and Experimental Alzheimer’s Research, Medical University of Innsbruck, Innsbruck, Austria

This is a well done contribution by a group having good experience in organotypic brain slice cultures. The MS is well written, the methods clear and the pictures of good quality. I have a few suggestions:

1. Please provide more details in the Abstract, especially, on the organotypic brain slice model (3xTg mice, chopper slices, 350 µm, postnatal d8-9, cultured for > 2weeks on 0.4 µm pore semipermeable membranes). Please also add this information in the Box research highlights. This is important to see on one view which kind of slices you mean.
2. I suggest that you are more careful about the used number of animals. In Abstract you write an estimated 20,000 mice per year for dementia research and on page 3, right col. sec. paragr. you write ... were used in AD research, and you write 20,000 transgenic; assuming from 30 mice per paper. What do you mean: (1) per year in UK, Europe or the world?, (2) total mice per year or only transgenic per year? (3) based on which fact you assume 30 mice per paper (value 30 is just your thoughts or is this based on an official reference?). I suggest that you check an EU report on the total number of animals/mice/transgenic, or you check your national institution (and also give the official reference). Such rough values could be very problematic.
3. Page 3 third paragr: .... importantly, slice cultures are prepared from neonatal mice ... this (neonatal) should be explained as it is unclear.
4. It would be helpful to give the average weight of the postnatal d8-9 mice. We usually weigh them, to be sure about the stage. Or do you have other rules to be sure on the correct stage. Also mention up to which age does your model work?
5. Page 5, point 6: typo: 2x remove
6. Page 7 right col, last paragr: ....when reporting on the phosphorylation of tau via LiCl and GSK-3, the authors may cite the recent paper Foidl and Humpel (2018) in Frontiers Aging Neurosci. on hyperphosphorylation of tau in organotypic brain slices induced by okadaic acid. In this respect also the review on brain culture slices by Humpel (2015) in Neuroscience should be cited (page 4, left col, line, 14).
7. Please explain better your definition of the n-number (page 8, right col.) .... one postnatal d8 or 9 pup can provide an n=36. I think that one mouse only gives an n=1; for an experiment you need at least 6-8 different mice (n=6-8); but one mouse can give up to 36 different treatments (or in duplicate 2x18). What is your suggestion on power calculations (how many slices per group?). Please explain better any statistical issues.
8. Finally, please state in your conclusion the limits of the brain slices; how to study genetic (familiar) versus sporadic AD, how comparable is the developmental stage with the adult stage (maturation of neurons after 2 weeks in culture?), how long do you need to culture to get at least an early comparable adult stage, what about axonal networks and the problem of axotomy.

**Are a suitable application and appropriate end-users identified?**

 Yes

**If applicable, is the statistical analysis and its interpretation appropriate?**

 Not applicable

**Are the 3Rs implications of the work described accurately?**

 Yes

I have read this submission. I believe that I have an appropriate level of expertise to confirm that it is of an acceptable scientific standard.

# Peer review: Referee response for version 1

**DOI:** 10.5256/f1000research.15785.r34021

**Published:** 2018-05-18

**Authors:** Claire S. Durrant

**Affiliations:** 1 John van Geest Centre for Brain Repair, Department of Clinical Neurosciences, University of Cambridge, Cambridge, UK

This well written method article describes the process of making whole brain organotypic slices from 3xTg-AD mice to use for studies of Alzheimer’s related pathology. The main focus of the paper is on how this method can be used to reduce and refine the use of animals in Alzheimer’s disease (AD) research. The methods of creating and maintaining the slice cultures are clearly described in detail allowing for future replication by other groups. Specific protocols for preparing slice material for immunostaining, immunoblotting and ELISA are also described effectively. The experimental results given from previously published work, as well as the cited work of others, serve as excellent examples of how this system can be utilised in AD studies with particular emphasis on the 3Rs. In particular, the authors highlight the ability to test multiple timepoints or multiple compounds on tissue from the same animal resulting in not only a reduction in the number of animals required, but also better controls for biological variability. I agree with the authors’ conclusions that brain slice models of Alzheimer’s disease can partly replace biochemical/cellular/molecular studies in older animals that have developed pathology.

 The authors provide a useful comparison between changes observed in human brain, aged 3xTgAD brain and brain slice cultures, with the raw data provided. Their observations that slice cultures show an accelerated phenotype when compared to adult mice is interesting and in agreement with what we have seen in hippocampal slices cultures from TgCRND8 mice (reference 9 in this paper). Future work seeking to elucidate why this is the case could be very informative.

 Whilst the methods in this paper have been previously described elsewhere, the detailed description of culture preparation, experimental design and methods for analysis in the context of AD are a useful and timely addition to the literature. The tips relating to assessment of culture health (a white slice becoming translucent) and issues to look out for that may harm the health of the slices (excess medium on the membrane insert) are completely aligned with our own experience and very useful for someone seeking to perform slice culture experiments for the first time. **I have no reservations in approving this manuscript.**

**Minor comments:**

- Whilst actin is a valid control protein in western blots, it could be argued that beta-III tubulin (or alternative neuronal markers) may be a more informative control when normalising synaptic protein levels. This controls for any difference in neuronal number between samples which may alter synaptic protein levels in the absence of a synaptic specific change. This would be more important in cases where synaptic protein levels are down, but there is also evidence for neuronal loss.
- It would be interesting to see whether the 3xTg-AD brain slices lose synaptic proteins if maintained for longer than 28 days in vitro. It may be that this phenotype appears after the observed changes in tau and Aβ in this culture system.
- It should be stated whether the 3xTg-AD mice used in this study are homozygous or heterozygous (I am presuming homozygous but this is not explicitly stated). It appears that the wild-type mice used as controls for the 3xTg-AD are background-matched, but not littermates. This is standard practice for many homozygous mouse lines but care should be taken to ensure backcrossing of the two lines to prevent genetic drift between colonies that have remained separated for a long time. Ideally, littermates would be used, but I understand that the homozygosity of this mouse model would complicate this and result in a greater number of animals being required to generate all genotypes. If separate colonies for transgenic and wild type pups are necessary, litters should ideally be synchronised to control for minor differences in culture preparation/ harvesting on different days.

**Are a suitable application and appropriate end-users identified?**

 Yes

**If applicable, is the statistical analysis and its interpretation appropriate?**

 Yes

**Are the 3Rs implications of the work described accurately?**

 Yes

I have read this submission. I believe that I have an appropriate level of expertise to confirm that it is of an acceptable scientific standard.
